# Anti-obesity activity of OBEX is regulated by activation of thermogenesis and decreasing adiposity gain

**DOI:** 10.1038/s41598-018-34840-7

**Published:** 2018-11-21

**Authors:** Marcos C. Carreira, Sara Andrade, Andrea Gonzalez-Izquierdo, Maria Amil, Cintia Folgueira, Mariana P. Monteiro, Eduardo Sanz, Ana B. Crujeiras, Felipe F. Casanueva

**Affiliations:** 1 Lab de Endocrinología Molecular, Instituto de Investigaciones Sanitarias de Santiago de Compostela (IDIS), Complejo Hospitalario de Santiago (CHUS), A Coruña, Spain; 2 CIBER Fisiopatología Obesidad y Nutrición (CIBERobn), Instituto de Salud Carlos III, Madrid, Spain; 3 Clinical and Experimental Endocrinology, Unit for Multidisciplinary Research in Biomedicine UMIB, Instituto de Ciências Biomédicas Abel Salazar (ICBAS), University of Porto, Porto, Portugal; 4 Department of Physiology, CIMUS, University of Santiago de Compostela-Instituto de Investigacion Sanitaria, A Coruña, Spain; 5 Grupo Fisiopatologia Endocrina, Instituto de Investigación Sanitaria de Santiago de Compostela, Complexo, Hospitalario Universitario de Santiago (CHUS/SERGAS), Santiago de Compostela, Spain; 6 Catalysis S.L., Madrid, Spain; 7 Universidad de Santiago de Compostela (USC), Santiago de Compostela, Spain

## Abstract

The incidence of obesity has been increasing dramatically worldwide over the past decades, thus requiring novel and effective therapeutic approaches. OBEX is an oral nutritional supplement composed of antioxidants with antiobesity activity. The effects of OBEX have been tested *in vivo* and *in vitr*o. *In vivo*, OBEX reduces weight gain by decreasing adiposity gain and increasing energy expenditure in high fat diet-fed mice through the activation of thermogenesis in brown adipose tissue (BAT) independent of eating behaviors. *In vitro* analysis with 3T3-F442A cells revealed anti-proliferative and anti-differentiation effects of OBEX. In addition, OBEX induced a clear reduction of the lipid load in mature adipocytes obtained from 3T3-F442A cells. Overall, our findings suggest that OBEX has a protective effect against an obesogenic environment.

## Introduction

The prevalence of obesity has increased during the last decades, reaching a global epidemic. According to the World Health Organization (WHO), in 2014, almost 2 billion adults were overweight and 600 million were obese^1^. In addition to the social implications, obesity is responsible for the development of many complications such as hypertension, heart disease, stroke, insulin resistance and type 2 diabetes, nonalcoholic fatty liver disease, and several types of cancer^2–4^. Moreover, the existing therapies for obesity, including nutritional intervention and exercise, are not completely effective and are often followed by weight regain^5,6^. For these reasons, new therapeutic approaches are necessary urgently for many patients. One such approach is OBEX, an oral nutritional supplement specifically prepared with natural antioxidants with recognized antiobesity effects. OBEX contains slimaluma, phaseolamina, L-carnitine, inulin, and açaí berry; several studies have shown that these components exert antiobesity effect and control metabolic parameters such as fasting glucose, cholesterol, and triglyceride levels^7–15^. In addition, it contains various essential amino acids, vitamins, and minerals. OBEX is currently being investigated in clinical trials as part of a possible treatment for overweight and obese patients with impaired fasting glucose to induce weight loss and ameliorate metabolic disturbances related to obesity and overweight. Although the mechanism of action of OBEX is unknown, a recent report has suggested the beneficial effect of OBEX on anthropometric measures and body composition in overweight and obese subjects^16^. On the basis of these results, we investigated the effects of OBEX on *in vivo* and *in vitro* models of obesity. Its antiweight gain and antiadipogenesis effects in mice fed a high-fat diet were evaluated. In addition, its action on the 3T3-F442A cell line was tested in terms of regulation of proliferation capacity and differentiation into mature adipocytes as well as its effects on mature adipocytes. In this context, OBEX could reduce the weight gain *in vivo* by decreasing fat mass gain without affecting food intake. In addition, on the 3T3-F442A cell line, OBEX inhibited proliferation of pre-adipocytes, blocked the differentiation of pre-adipocytes into new adipocytes, and decreased the lipid load in mature adipocytes.

## Results

### OBEX treatment reduces body weight gain and adiposity independent of food intake in mice fed with high fat diet (HFD)

We fed mice a diet of 60% fat ad libitum and treated them daily with 0.10, 0.25, or 0.50 g/day of OBEX in drinking water while monitoring body weight gain. No significant differences in daily water consumption were observed between the different groups of mice treated with OBEX (Supplementary Information Fig. 1).

**Figure 1:**
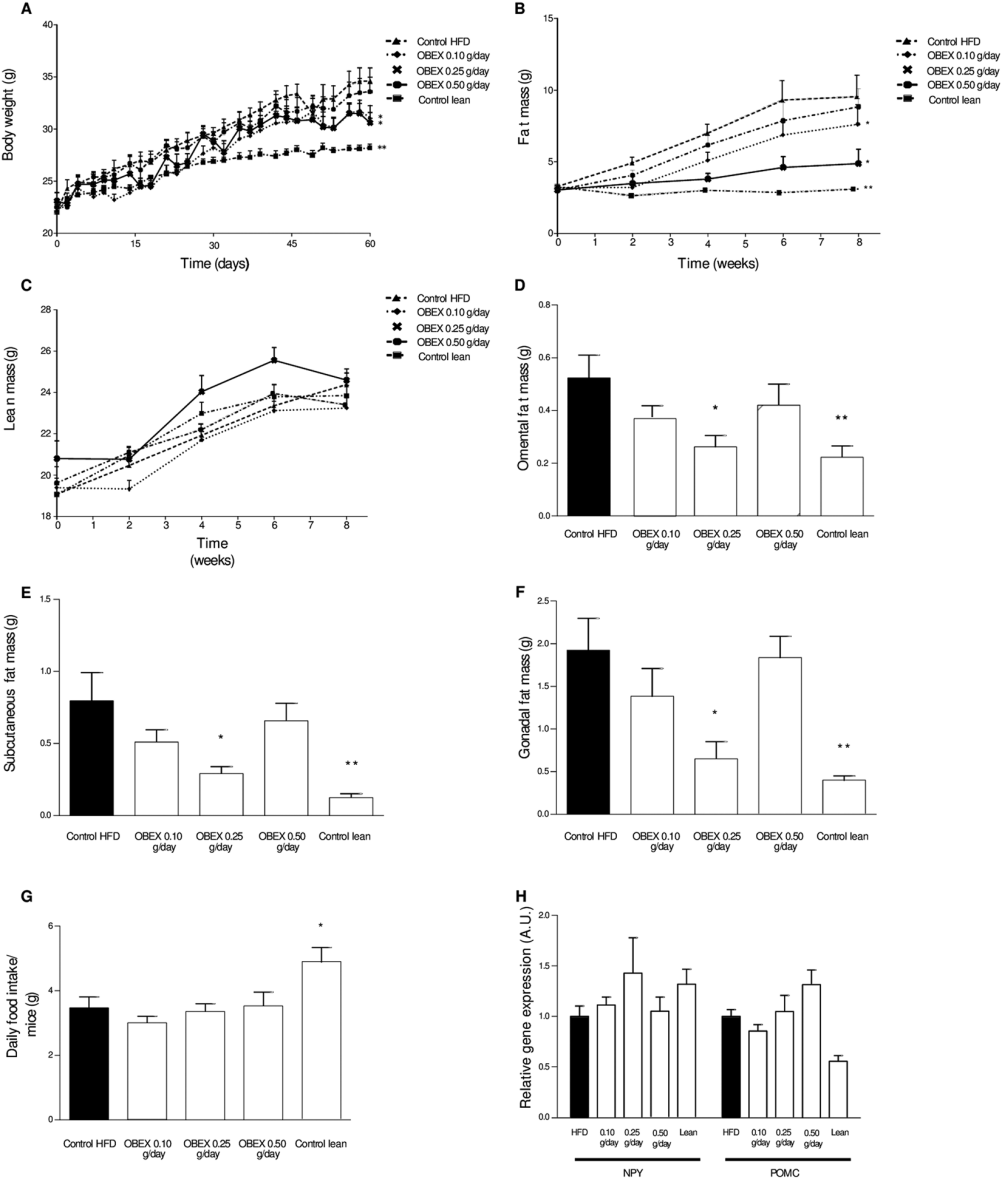
Effect of 8 weeks of treatment with OBEX (0.10, 0.25, and 0.50 g/d) on body weight evolution (**A**), fat mass change (**B**), and lean mass change (**C**) in mice fed a HFD *ad libitum*. Effect of 8 weeks of treatment with OBEX (0.10, 0.25, and 0.50 g/d) on postmortem omental (**D**), subcutaneous (**E**), and gonadal (**F**) fat mass. Effect of 8 weeks of treatment with OBEX on daily food intake (**G**) and relative gene expression of NPY and POMC (**H**). Data are represented as mean and SEM, n = 10 animals/group. *P < 0.05, **P < 0.01 vs. the HFD group.

At the end of the experiment, the animals that received the 0.25 g/day and 0.10 g/day doses of OBEX gained significantly less weight than the HFD control animals; the 0.25 g/day dose was the most effective with a 12% less weight gain, followed by the 0.10 g/day dose with 10% less weight gain (Fig. 1A). However, the 0.50 g/day dose did not appear to block the weight gain induced by the HFD (Fig. 1A). In parallel with the decreased weight gain, fat mass gain was also significantly lower in the OBEX-treated animals than in the HFD control group (Fig. 1B). As expected, the average lean mass throughout the experimental time presented fewer and not statistically significant differences between the groups (Fig. 1C). Post-mortem analysis confirmed a significant decrease in omental (Fig. 1D), subcutaneous (Fig. 1E), and gonadal (Fig. 1F) fat mass. Despite the marked differences found in the body weight of OBEX-treated animals, these differences could not be translated to the food intake of the different groups (Fig. 1G). In addition, the expression levels of the orexigenic neuropeptide Y (NPY) gene and the anorexigenic proopiomelanocortin (POMC) genes were evaluated in the hypothalamus, and no significant differences were found between the groups for both the genes (Fig. 1H).

### OBEX treatment increases energy expenditure and BAT thermogenesis in mice fed with HFD

**Figure 2:**
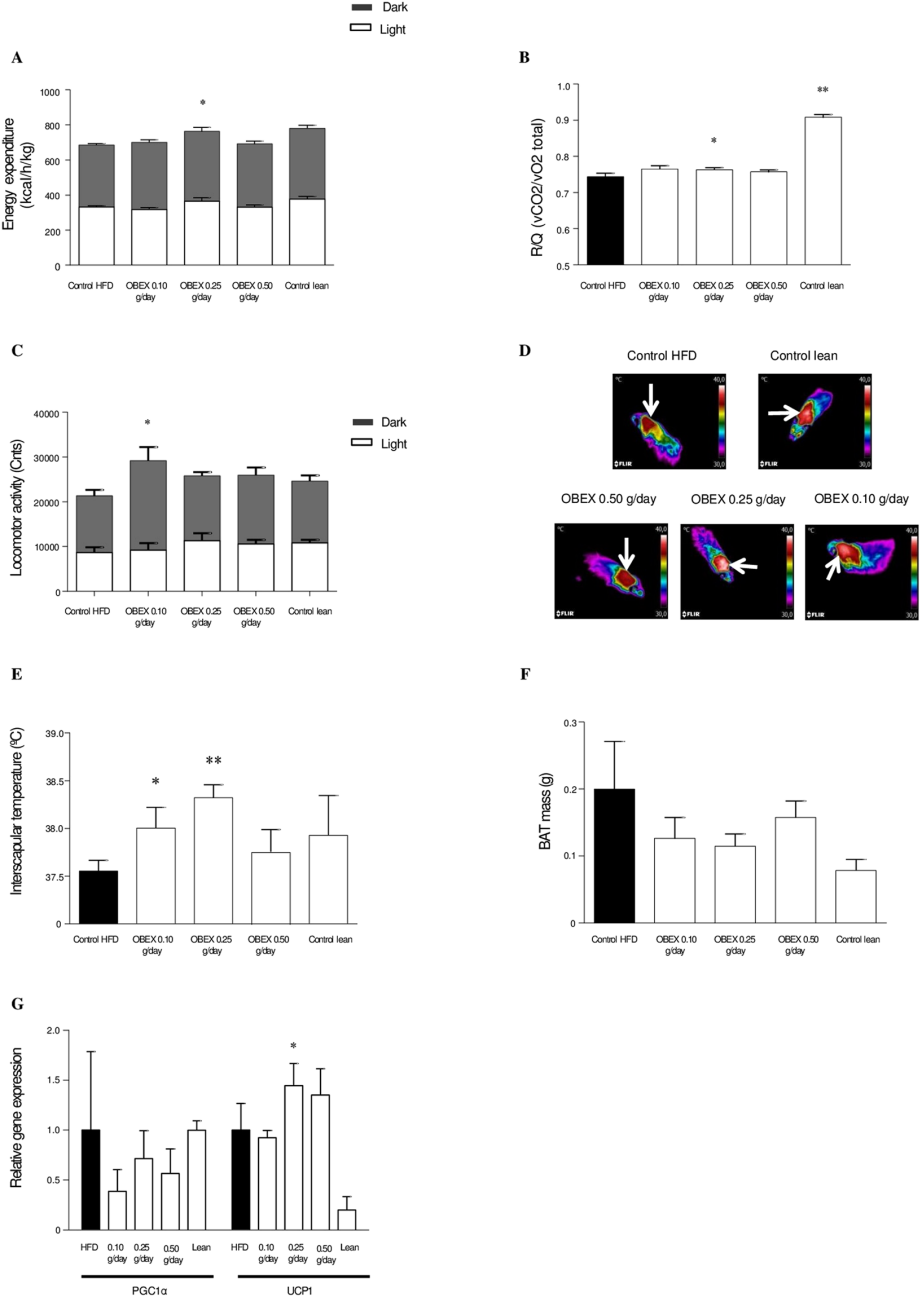
Effect of 8 weeks of treatment with OBEX (0.10, 0.25, and 0.50 g/d) on energy expenditure (**A**), respiratory quotient (**B**), locomotor activity (**C**), interscapular temperature (**D**), BAT mass (**E**), and BAT gene expression of UCP1 and PGC1α (**G**) in mice fed a HFD *ad libitum*. Pictures are representative images of each experimental group (×20 magnification). Data are represented as mean and SEM, n = 10 animals/group. *P < 0.05, **P < 0.01 vs. the HFD group.

Because the effect of OBEX appears to be independent of food intake, we reasoned that the weight loss was due to increased energy expenditure. Thus, we used metabolic cages to monitor energy expenditure in HFD-fed mice treated with OBEX. In agreement with the decreased weight gain, the OBEX 0.25 g/day group showed a significant increase in energy expenditure (Fig. 2A) and respiratory quotient (RQ) (Fig. 2B) when compared with the control group, whereas the locomotor activity (Fig. 2C) remained unchanged. Next, we investigated whether BAT thermogenesis was responsible for the increased energy expenditure. There were no differences in rectal body temperature (data not shown); however, the interscapular temperature recorded using an infrared camera (Fig. 2D) was significantly higher in the OBEX 0.25 g/day group (38.4 ± 0.22 °C) than in the HFD control groups (37.55 ± 0.11 °C) (Fig. 2E). BAT analysis showed no significant differences in mass between the groups (Fig. 2F), but showed that the gene expression level of the main thermogenic biomarker uncoupling protein 1 (UCP1) was significantly increased in the BAT of HFD mice treated with 0.25 g/day of OBEX (Fig. 2G). However, no alterations were observed in the expression levels of other thermogenic biomarkers such as peroxisome proliferator-activated receptor γ coactivator 1-α (PGC1α). (Figure 2G).

### OBEX treatment reduces lipid load and changes the gene expression profile in the liver of mice fed with HFD

**Figure 3:**
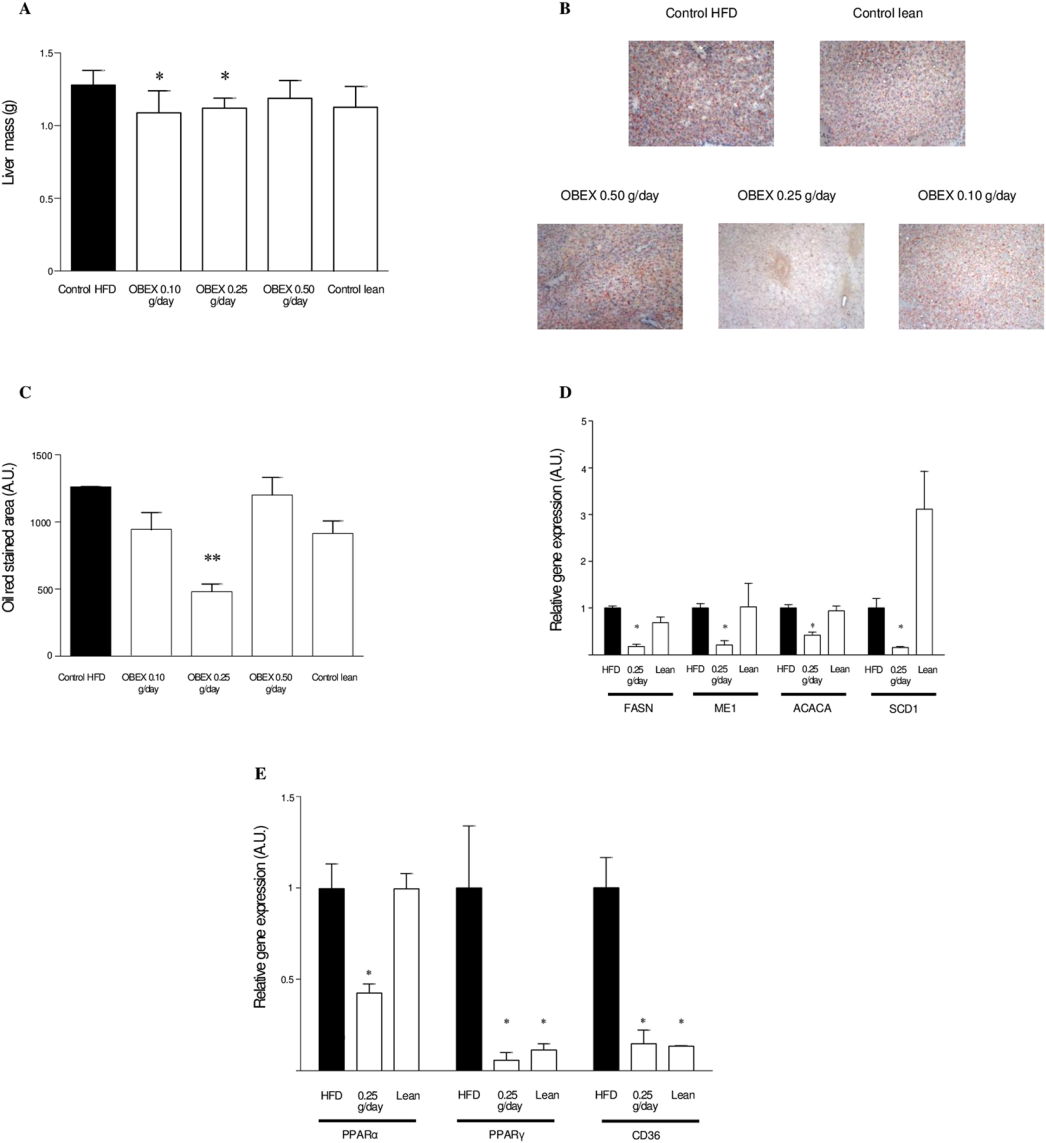
Effect of 8 weeks of treatment with OBEX (0.10, 0.25, and 0.50 g/d) on total liver weight (**A**) and lipid content shown as oil red staining (×20 magnification) (**B**) and oil red staining quantification (**C**) in mice fed a HFD *ad libitum*. Effect of 8 weeks of treatment with OBEX (0.25 g/d) on the expression of the liver genes FASN, ME1, ACACA, and SCD1 (**D**) and PPARα, PPARγ, CD36, FABP4, and LPL (**E**) in mice fed a HFD *ad libitum*. Pictures are representative images of oil-red stained liver for each experimental group. Data are represented as mean and SEM, n = 10 animals/group. *P < 0.05, **P < 0.01 vs. the HFD group.

Livers from mice fed with HFD and treated with OBEX for 8 weeks were examined postmortem. Hepatomegaly observed in HFD-fed mice was reversed by OBEX at doses of 0.25 g/day and 0.10 g/day, with 12.5% and 15% reduction in liver weight, respectively (Fig. 3A). In addition, lipid content in the liver was reduced by more than 60% in the mice treated with OBEX at 0.25 g/day (480.30 ± 57.70 arbitrary units (A.U.)) compared with the HFD control group (1262.70 ± 0.20 A.U.) (Fig. 3B,C). To further investigate hepatosteatosis, we analyzed the expression of key metabolic genes in the liver. OBEX treatment reduced the expression of key lipogenic genes such as fatty acid synthase (FASN), malic enzyme (ME1), acetyl-CoAcarboxylase (ACACA), and stearoyl-CoA desaturase 1 (SCD1) in the HFD-fed group compared to that in the HFD control group (Fig. 3D). The expression of genes involved in increasing lipid load, such as peroxisome proliferator-activated receptor alpha and gamma (PPARα, PPARγ), and fatty acid translocase (CD36) (Fig. 3E), was also reduced, whereas the expression of other genes implicated in lipid load, such as fatty acid binding protein 4 (FABP4) and lipoprotein lipase (LPL), remained unchanged (data not shown). In addition, OBEX treatment did not change the expression of gluconeogenic (glucose-6-phosphatase (G6PC) and phosphoenolpyruvate carboxykinase (PCK)) or glycolytic (Pyruvate kinase (PK1R) and glucokinase (GCK) genes (data not shown).

### OBEX treatment reduces circulating lipid profile of mice fed with HFD

**Figure 4:**
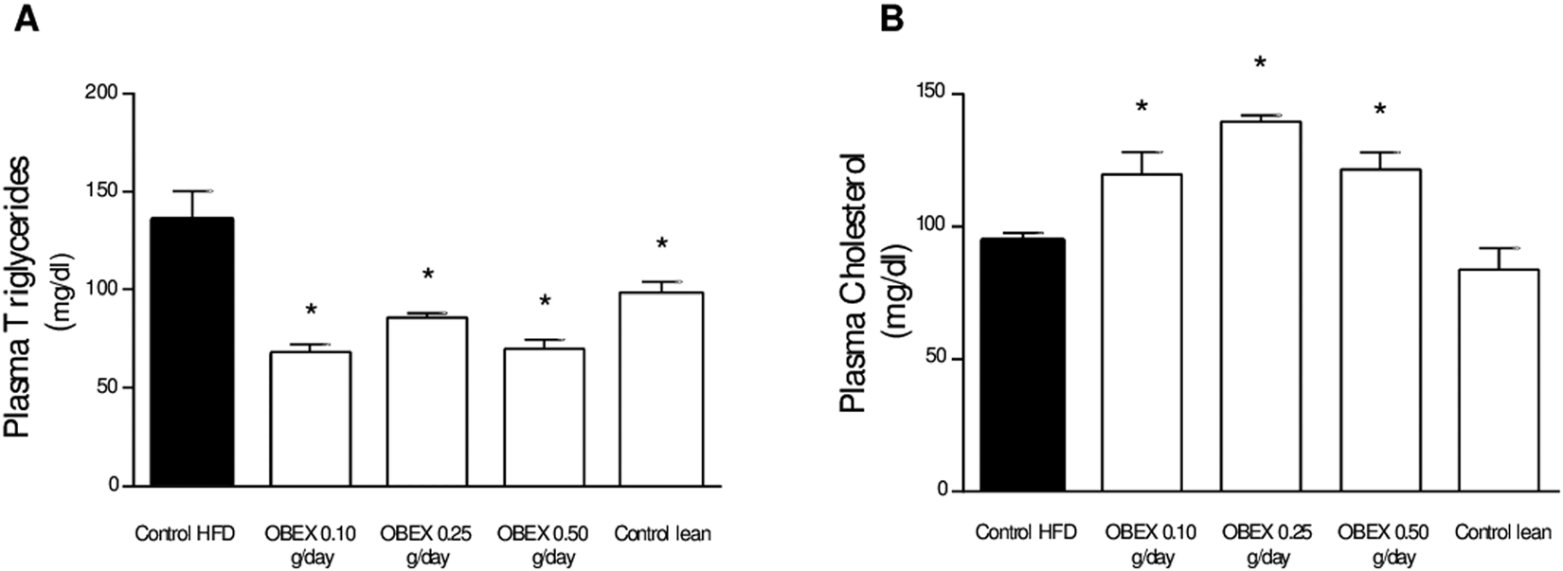
Effect of 8 weeks of treatment with OBEX (0.10, 0.25, and 0.50 g/d) on plasma triglycerides (**A**) and total cholesterol (**B**) levels in mice fed a HFD *ad libitum*. Data are represented as mean and SEM, n = 10 animals/group. *P < 0.05 vs. the HFD group.

Mice treated with OBEX concurrently with HFD showed a marked decrease of approximately 60% in circulating triglycerides (TGA) compared to that in the HFD control group (Fig. 4A), and were equivalent to TGA levels observed in lean control animals. In addition, plasma from animals treated with OBEX showed a significant increase in the plasma total cholesterol (Fig. 4B).

### OBEX administration regulates the process of adipogenesis in the 3T3-F442A cell line

**Figure 5:**
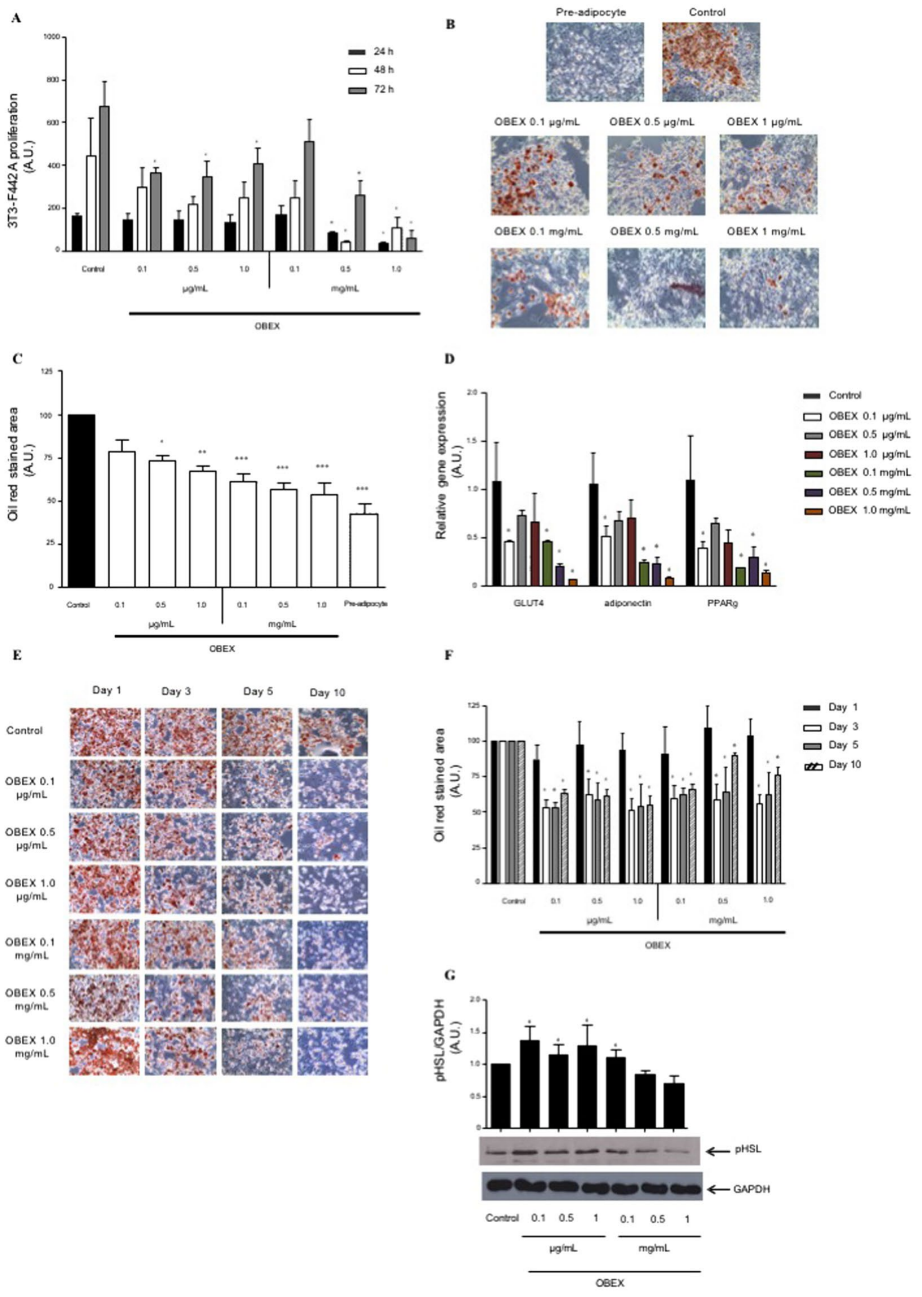
Effect of increasing concentrations of OBEX on the proliferation of 3T3-F442A cells during 24, 48, and 72 h of treatment (**A**). Effect of increasing concentrations of OBEX on differentiation of 3T3-F442A into adipocytes during 10 days represented as oil red-stained image (×20 magnification) (**B**), quantification of oil red staining (**C**), and relative gene expression of differentiation markers GLUT4, PPARγ, and adiponectin (**D**). Pictures are representative images of oil-red staining for each experimental group. Effect of increasing concentrations of OBEX on mature adipocytes differentiated from 3T3-F442A cells for 1, 3, 5, and 10 days shown as oil red-stained image (×20 magnification) (**E**) and quantification (**F**) and protein level of pHSL for 3 days (the image represents two different blots: pHSL and GAPDH (control)) (**G**). Data are represented as mean and SEM, n = 3–4 experiments in triplicate. *P < 0.05, **P < 0.01 and ***P < 0.001 vs. control.

To evaluate a possible direct effect of OBEX at the cellular level in the adipose tissue, the *in vitro* effect of OBEX was evaluated on the 3T3-F442A cell line. The cells were cultured in the presence of increasing doses of OBEX diluted in the culture medium, and their proliferation was evaluated with WST-1 assays on different days of treatment. OBEX decreased the proliferation of 3T3-F442A pre-adipocytes at all concentrations used, except 0.1 mg/mL, at the incubation time of 72 h (Fig. 5A). At higher doses, the inhibitory effect on proliferation was already observed at shorter incubation times of 48 and even at 24 h (Fig. 5A).

We next investigated the influence of OBEX on differentiation of 3T3-F442A cells into adipocytes. The presence of OBEX diluted in the medium during the activation of adipogenesis decreased the oil red staining of the newly converted adipocytes (Fig. 5B,C). In parallel, the expression levels of differentiation markers such as GLUT 4, PPARγ, and adiponectin (Fig. 5D) were significantly decreased in comparison with non-treated adipocytes.

Finally, we tested the effect of OBEX on a population of mature adipocytes differentiated from 3T3-F442A cells. The same increasing concentrations of OBEX were maintained in the culture medium for up to 10 days. From day 3, all the concentrations of OBEX produced a progressive reduction in oil red staining (Fig. 5E,F). In addition, during the treatment, the expression levels of differentiation markers GLUT 4, PPARγ, and adiponectin were constant compared with that in the nontreated controls (data not shown). Accordingly, with the strong reduction in lipid load in mature adipocytes following treatment with OBEX from day 3, protein levels of phosphorylated hormone sensitive lipase (pHSL), the main indicator of lypolysis, were significantly increased at day 3, with higher activation at the lower concentration of OBEX (0.1 µg/mL) (Fig. 5G).

## Discussion

In this work, we performed an observational study of the antiobesity effect of OBEX using both *in vivo* and *in vitro* models. We showed that OBEX administration in HFD-fed mice reduces weight gain by decreasing adiposity gain and increasing energy expenditure and thermogenesis independent of feeding behavior. In addition, in the 3T3-F442A cell line, OBEX could decrease the proliferation of precursors, the differentiation process, and lipid load in mature adipocytes, suggesting an eventual effect of decrease in fat mass.

OBEX is a nutritional supplement that includes antioxidant compounds in its composition and opens new opportunities for its use as an obesity preventive agent both i*n vivo* and for future patients. In this sense, we have not used a model of obesity, but a model that combined HFD with OBEX.

OBEX induced a reduction in weight gain of C57BL/6 mice fed with HFD. Animals receiving the two lower concentrations of OBEX gained significantly less weight than the HFD control animals. The 0.25 g/day dose was the most effective, with a 12% less weight gain, followed by the 0.10 g/day dose, with 10% less weight gain. However, the higher dose of OBEX, 0.50 g/day, had no effect on body weight. We suggest that it may be due to a dose-saturation effect or a phenomenon of tachyphylaxis i.e., the gradual decrease in the effect of a drug when given continuously, repeatedly, or at high doses. Currently, the guidelines for pharmacologic treatment of obesity consider that a drug is effective if the weight loss is equal or superior to 5% during the first year of treatment^17^. OBEX contains slimaluma, an extract from the *Caralluma fimbriata* plant, which is associated with hunger suppression^13,14^; however, in the present study, OBEX treatment did not show effects on food intake compared to that in the HFD control groups. Interestingly, the analysis of body composition showed that OBEX-treated animals gained less fat mass that the HFD control group, and lean mass remained constant, suggesting that this effect occurred exclusively at the expense of fat mass. Our findings suggest that the mechanism by which OBEX reduces body weight gain involves increased energy expenditure through the activation of BAT thermogenesis. In addition to playing a key role in thermogenesis activation, OBEX can also modulate peripheral lipid metabolism, because liver metabolism was altered together with white adipose tissues (WAT) depots.

At this point, the specific mechanism by which OBEX produces the beneficial effects in our rodent model remain to be completely elucidated. In addition, OBEX is a product containing several compounds in its formulation, thereby making it difficult to isolate the molecular mechanism by which it exerts its effects and mimic it. This study describes for the first time that the administration of OBEX reduces body weight and adiposity in mice fed a HFD independent of food intake through the promotion of BAT thermogenesis. Many peripheral signals are attributed to the regulation of energy expenditure through neuronal mechanisms, and there are some evidence indicating that BAT activity as well as promotion of browning phenomenon in WAT are regulated by the central nervous system^18–22^. In this sense, at the present, we cannot conclude that any of the components of OBEX can act on the BAT directly or through a central mechanism. In addition, a possible thyroid hormone-like activity of the compound was excluded, because serum rodent-specific thyroid-stimulating hormone (TSH) values were not altered (data not shown).

The reduction in fat gain observed after treatment with OBEX compared to the HFD control group suggested the idea of a direct effect of OBEX on adipocytes. Adipocytes are involved in the maintenance of energy balance and lipid homeostasis by releasing free fatty acids and storing triacylglycerols in response to changes in general energy demands. In environments of positive energy balance, increased energy is stored by increasing the content of the adipose tissue. Pre-adipocytes are the precursors of the adipocytes that are being continuously produced through the process of adipogenesis^23,24^. The dysregulation of the processes of proliferation of pre-adipocytes (hyperplasia) and adipogenesis (hypertrophy) may determine the amount of increase in the adipose tissue or the size of mature adipocytes contributing to the development of obesity^25,26^. Another very important aspect that requires attention is the effect of OBEX on 3T3-F442A cells, a well-characterized model for studying the differentiation of white adipocytes^27^. We found that OBEX could regulate adipocytes in a three-way manner. First, OBEX treatment showed an anti-proliferative capacity on pre-adipocytes. This means that there was a smaller pool of precursors capable of becoming mature adipocytes. Second, OBEX reduces the adipogenic capacity of existing pre-adipocytes, resulting in a smaller number of mature adipocytes. Third, OBEX reduces the lipid load of mature adipocytes, thus implying a lower fat storage capacity. Interestingly, the three observed effects were produced using the same concentration of OBEX and at relatively low doses; this suggests that this triple effect could occur simultaneously *in vivo*.

Furthermore, these data show that the regulation of proliferation/differentiation of the adipocytes, as well as the mature adipocytes themselves, increases the chances of success for a weight reduction treatment. From an integrative point of view, our data suggest that OBEX causes a decrease in the amount of total fat mass. With these results, it can be argued that an insufficient amount of adipose tissue results in an insufficient storage of lipids, inducing high levels of circulating lipids with its consequent accumulation in liver or muscle and leading to insulin resistance and diabetes^26^. However, this idea does not seem the most plausible considering the data obtained *in vivo*. Mice treated with OBEX and fed a HFD did not show fatty liver, and a marked decrease in lipid levels compared to the HFD control group was observed. Moreover, the circulating lipid levels in the treated group were lower than those in the HFD control group. The total cholesterol levels alone were higher than those in the control animals, which could be explained given the general improvement that could increase the fraction of high-density lipoproteins (HDLs) versus low-density lipoproteins (LDLs). Therefore, it is suggested that OBEX in obesogenic environments protects against the development of fatty liver and obesity.

In summary, the present study shows that OBEX decreases body weight gain and adiposity in mice fed with a HFD independent of food intake through BAT thermogenesis. The results presented here show that OBEX modifies the proliferation of pre-adipocyte precursors, affecting the process of adipogenesis, and has a direct effect on adipocytes, the main cells responsible for energy storage. This mechanism could imply the existence of a new regulator of the amount of total adipose tissue whose molecular mechanism remains unknown, but could possibly lead to the development of anti-obesity therapies. Although our results have opened new and interesting issues for future investigations, other issues remain to be elucidated. Specifically, further studies are required to reveal the exact molecular mechanisms through which OBEX affects adipogenesis and obesity.

## Experimental Procedures

### Study product

The supplement OBEX used in this work is marketed as a food supplement by Catalysis Laboratories. The bags of OBEX (4 g) contained the ingredients specified in Table 1 (Supplementary Information).

### Animals

Fifty male, 8-weeks old C57BL6/J normal weight mice (with an initial weight of 20–25 g), from the central animal facilities of the Universidad de Santiago de Compostela were maintained in controlled conditions of temperature, humidity, and illumination (12-h controlled photoperiod). They were allowed to acclimatize for 1 week on arrival. After the acclimatization period, the animals were randomized into five weight-matched groups (n = 10/group). One group had *ad libitum* access to a standard diet (SAFE- Panlab, Spain), with 5.5% lipid, 23% protein, and 70% carbohydrate content. The other four groups were fed with HFD (Open Source Diets, Research Diets; Brogaarden, Denmark, Reference D 12492) with 60% lipid, 20% protein, and 20% carbohydrate. Of the HFD groups, the control group had access to water, and the others were treated with OBEX in three different concentrations (0.10, 0.25, and 0.50 g/day) daily diluted in the drinking water during all the experiment. Body weight, food, and water intake were measured during the experimental period. Finally, the animals were euthanized with CO_2_ and decapitated, and the organs and tissue were removed rapidly, immediately frozen on dry ice, and kept at −80 °C until analysis. All animal experiments and procedures involved in this study were approved by the Ethical Committee at the University of Santiago de Compostela, in accordance with the European Union Normative for the use of experimental animals.

### Food intake

The weight of the food provided to the animals was determined daily for a single cage. Daily food consumption was calculated from a 3-day average.

### Body composition and indirect calorimetry

Body composition studies were performed every 2 weeks using a nuclear magnetic resonance imaging system (MRI) (Whole Body Composition Analyser, EchoMRI, Echo Medical Systems, USA). Mice were analyzed for energy expenditure, respiratory quotient, and locomotor activity by using a 32-cage calorimetry system (LabMaster; TSE System)^28,29^. The instrument is a combination of highly sensitive feeding and drinking sensors for automated measurement. The calorimetry system determines O_2_ consumption, CO_2_ production, and RQ (*V*co_2_/*V*o_2_). A photobeam-based activity-monitoring system detects and records every movement including rearing and climbing movements in every individual cage. Detection of animal location is performed with infrared sensor pairs arranged in strips for horizontal and vertical activity. All the parameters can be measured continuously and simultaneously in up to 32 cages.

### Core body temperature and interscapular temperature

Body temperature and interscapular temperature data were acquired the day before sacrifice. To measure body temperature, the temperature was measured rectally using a thermometer (BAT-12 Microprobe-Thermometer). To measure the interscapular temperature, the hair in this area was previously shaved before taking a thermogenic photograph (E60bx: Compact-Infrared-Thermal-Imaging-Camera; FLIR). Images were analyzed using specific FLIR-Tools software^29^.

### RNA extraction and quantitative RT-PCR

Total RNA extraction was performed using the GeneJET RNA Purification Kit (Thermo Scientific, Spain) according to the manufacturer’s instructions. The RNA (500 ng) was retrotranscribed into cDNA using the High Capacity cDNA Reverse Transcription Kit (Applied Biosystems, USA). The expression of the genes of interest was analyzed using TaqMan real-time PCR in Step One Plus system (Applied Biosystems, USA) with specific primers and probes obtained from inventoried TaqMan Gene, Expression Assays (Applied Biosystems, USA). All reactions were performed using the following cycling parameters: 50 °C for 2 min, 95 °C for 10 min, followed by 40 cycles of 95 °C for 15 s, 60 °C for 1 minute. For data analysis, the RNA level of the gene of interest was normalized using the β-actin values, according to the 2^−ΔΔCt^ method.

### Liver oil red staining

Staining of the liver was performed as previously described^30^. A portion of the mouse liver was removed from the livers of the euthanized mice, and rapidly frozen in Tissue-Tek® O.C.T. Compound (USA) in dry ice for oil red staining. The tissue was sectioned at 12 µm thickness. The sections were allowed to dry at room temperature for 10 min and were frozen at −80 °C. The following day, the oil red stock was prepared by adding 0.625 g of oil red to 100 mL of 100%. The working solution was then prepared by adding 1.5 parts of oil red to 1 part of deionized water, and the final solution was filtered with a 0.2-µm filter. The slides were removed from the freezer and allowed to equilibrate at room temperature for 10 min. The sections were submerged in oil red working solution for 10 min. The sections were counterstained with hematoxylin by submerging the section in hematoxylin for 15 s, and then rinsed under running tap water for 30 min. The slides were mounted with Aquatex® (Merck, Germany). Ten photos per section were captured with a bright light microscope with a total amplification of 200×. Images were analyzed using an image processing software (ImageJ v1.48, National Institutes of Health, USA) with a color deconvolution plug-in that separates the stained area from the initial image, allowing the quantification of the percentage of the area specifically stained.

### Plasma lipid analysis

For the quantitative determination of cholesterol and triglycerides in plasma, the Advia Chemistry Systems (Siemens Healthcare Diagnostics Inc.) was used according to the manufacturer’s instructions and using an ADVIA 2400 Chemistry System spectrophotometer.

### Protein extraction and western blot analysis

Cells were homogenized in cold RIPA buffer containing 200 mmol/L Tris/HCl (pH 7.4), 130 mmol/L NaCl, 10% (v/v) glycerol, 0.1% (v/v) SDS, 1% (v/v) Triton X-100, and 10 mol/L MgCl2 with antiprotease and antiphosphatase cocktail (Sigma-Aldrich, USA). The tissue lysates were centrifuged for 30 min at 18,000 *g* in a microfuge at 4 °C. Total protein lysates from cells (20 µg) were run on 10% sodium-dodecyl sulfate polyacrylamide (SDS-PAGE) and electroblotted onto a nitrocellulose membrane. The membranes were blocked for 1 h in 5% bovine serum albumin (BSA, Sigma-Aldrich, USA) and successively probed with primary antibodies overnight at 4 °C. For protein detection, we used horseradish peroxidase-conjugated secondary antibodies for 1 h at room temperature. Specific antigen–antibody bindings were visualized using reactive chemiluminescence detection according to the manufacturer’s instructions (Pierce ECL Western Blotting Substrate; Thermo Scientific). Image J software (NIH, USA) was used to quantify the volumes of specific bands. Data are expressed as percentages corrected for GADPH (A.U.).

Primary rabbit anti-pHSL (dilution 1:1000) (Ser660, catalog number 4126) polyclonal antibody was purchased from Cell Signaling (USA) and primary anti-GAPDH (dilution 1:5000) (catalog number AM4300) was purchased from Life Technologies Ltd (U.K). We used goat anti-rabbit secondary antibody (dilution 1:10000) (catalog number 31460) from ThermoFisher Scientific (USA) for pHSL and goat anti-mouse secondary antibody (dilution 1:10000) (catalog number 31430) from ThermoFisher Scientific (USA) for GAPDH.

### 3T3-F442A cell culture and differentiation

3T3-F442A cells (Sigma-Aldrich) were cultivated and maintained in Dulbecco’s modified Eagle medium (DMEM) (Lonza, Sapin) supplemented with 10% fetal bovine serum (FBS) (Lonza, Spain) and 1% penicillin-streptomycin (Lonza, Spain). Once they reached confluence, 3T3-F442A pre-adipocytes were differentiated into mature adipocytes. The cells were serum-deprived overnight, after which the cells were maintained in the first differentiation stimuli containing DMEM supplemented with 10% FBS, 1% penicillin-streptomycin, 0.5 mM 3-isobutyl-1-methylxanthine (IBMX) (Sigma-Aldrich, USA), 25 µM dexamethasone (Sigma-Aldrich, USA), and 5 µg/mL insulin (Sigma-Aldrich, USA) in presence or absence of OBEX at different doses for 3 days. In the next 7 days, the cells were maintained in DMEM media with 10% FBS, 1% penicillin-streptomycin, and supplemented with 1 µg/mL insulin in the presence or absence of OBEX at different doses. Differentiation of 3T3-F442A cells into adipocytes was estimated by morphologic analysis under light microscope, oil red staining, and reverse transcriptase-polymerase chain reaction (RT-PCR). Oil red staining was performed as described previously^31^. Oil red was dissolved by isopropanol and quantified by a spectrometer. For RT-PCR, differentiation markers such as GLUT 4, the insulin-regulated glucose transporter found primarily in the adipose tissue; adiponectin, an adipose tissue-secreted hormone; and PPARγ, a nuclear receptor that activates genes that stimulate lipid uptake and adipogenesis by fat cells, were evaluated. The effect of OBEX on adipocytes was analyzed as follows: once the adipocytes have been differentiated *in vitro* by the usual 3 + 7 day process described above (in the absence of OBEX), OBEX is added to the different doses indicated in the culture medium (DMEM + 10% FBS + 1% penicillin-streptomycin) for 1,3,5, or 10 days renewing the culture medium every 2 days.

### Proliferation studies

3T3-F442A cells were seeded in a 96-well microplate at the concentration of 1000 cells/well with DMEM, 10% FBS, and 1% penicillin-streptomycin. After 24 h, the cells were serum deprived for 6 h and then treated with OBEX in the presence of 10% FBS. At 24, 48, or 72 h after treatment, 10 µL of WST-1 reagent (Roche, Switzerland) was added to each well, and the microplates were incubated for 4 h at 37 °C. After incubation, the absorbance of each well was measured at 450 nm in an enzyme-linked immunosorbent assay (ELISA) microplate reader. Results were normalized with the value of absorbance for each medium + WST-1 without cells.

### Statistical analysis

Data analysis was performed using GraphPad Prism (version 4.0). Statistical analysis was conducted using one-way analysis of variance (ANOVA) followed by a post hoc correction. Results are shown as mean ± standard error (mean ± SE), unless otherwise specified. A p value of <0.05 was considered statistically significant.

## Electronic supplementary material

Supplementary information
