# Supplementary material for: Anti-obesity activity of OBEX is regulated by activation of thermogenesis and decreasing adiposity gain
Source: Sci Rep. 2018 Nov 21;8:17155. doi: 10.1038/s41598-018-34840-7 (PMC6249269; doi:10.1038/s41598-018-34840-7)
Supplement: Supplementary file 1 — Supplementary information [file 41598_2018_34840_MOESM1_ESM.pdf]

Anti-obesity activity of OBEX is regulated by activation of thermogenesis and decreasing adiposity gain

Marcos C. Carreira, Sara Andrade, Andrea Gonzalez-Izquierdo, Maria Amil, Cintia Folgueira, Mariana Monteiro, Eduardo Sanz, Ana B. Crujeiras, Felipe F. Casanueva

Table1. Ingredients found in the bags of OBEX (4g).

Ingredient	Amount
Caralluma Fimbriata	1500 mg
Inositol	500 mg
Choline	200 mg
Methionine	200 mg
Arginine	144 mg
Phaseolous Vulgaris	100 mg
Inulin	100 mg
Acai Berry	50 mg
Ornitine	50 mg
Carnitine Fumarate	13 mg
Zinc Sulfate	7 mg
Calcium Pantothenate	3 mg
Piridoxine	0,9 mg
Folic acid	0,1 mg

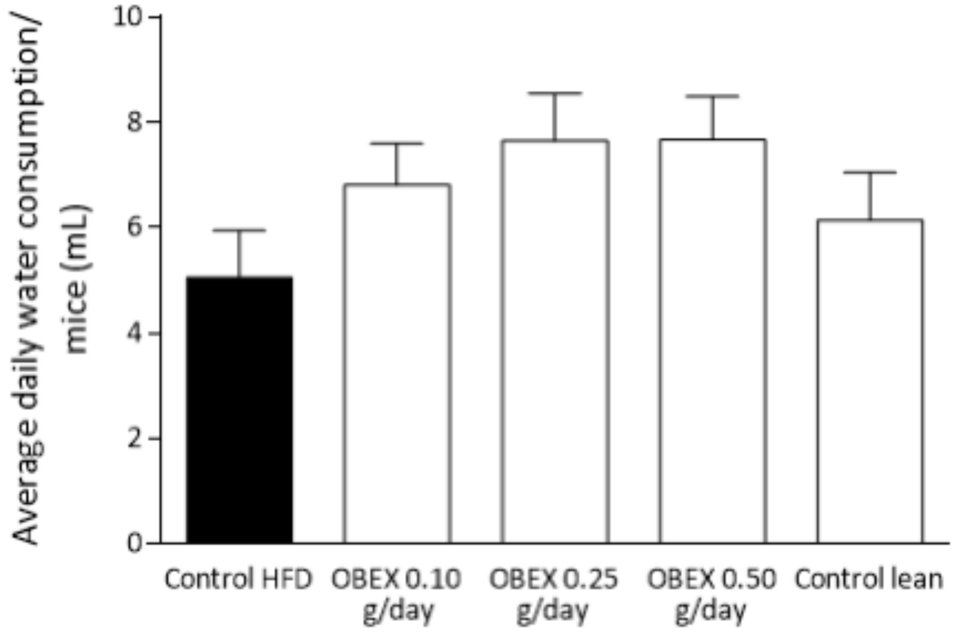

Supplementary Figure 1. Average daily water consumption per group of mice treated with OBEX and controls.
